# PTPN2/PTPN1 inhibition enhances *ex vivo* T- cell responses and potentiates mosunetuzumab activity in chronic lymphocytic leukemia

**DOI:** 10.3389/fimmu.2026.1948967

**Published:** 2026-10-08

**Authors:** Liron Yitzhak, Yamit Shorer Arbel, Yotam Bronstein, Ben-Zion Katz, Yair Herishanu

**Affiliations:** 1 Department of Hematology, Tel Aviv Sourasky University Medical Center (Ichilov), Tel Aviv, Israel; 2 Gray Faculty of Medicine and Health Sciences, Tel Aviv University, Tel-Aviv, Israel

**Keywords:** B cells, bispecific antibodies, CLL, PTPN1/PTPN2, T cells

## Abstract

**Background:**

Chronic lymphocytic leukemia (CLL) is characterized by T-cell dysfunction that limits the efficacy of immunotherapies, including CD20×CD3 bispecific antibodies. Protein tyrosine phosphatases non-receptor type 2/1 PTPN2/PTPN1 are central intracellular negative regulators of T-cell activation. Preclinical studies show that deletion or PTPN2/PTPN1 inhibition increased T-cell activation, proliferation, and antitumor activity. This study aimed to investigate the potential of PTPN2/PTPN1 inhibition in combination with the bispecific antibody mosunetuzumab as a therapeutic approach for CLL.

**Methods:**

Peripheral blood mononuclear cells (PBMCs) obtained from CLL patients were used in all experiments. The effects of PTPN2/PTPN1 inhibition in combination with mosunetuzumab, were evaluated using flow cytometry, confocal microscopy, ELISA, and Incucyte Live-Cell Analysis to assess T-cell activation, immune synapse formation and cytotoxic activity.

**Results:**

Pretreatment with the PTPN2/PTPN1 inhibitor ABBV-CLS-484 (AC484) enhanced T-cell activation following CD3/CD28 stimulation, as demonstrated by increased expression of the activation markers CD69 and CD25. Confocal microscopy revealed increased CD8+ T-cell size, enhanced granzyme B expression and release, and more robust immune synapse formation following treatment with mosunetuzumab +/-AC484. Incucyte imaging of CLL-T-cell co-cultures demonstrated enhanced T-cell proliferation, increased CLL-T-cell clustering, and a modest increase in cytotoxicity with the combination of AC484 and mosunetuzumab. Consistent with these findings, ELISA assays showed increased secretion of granzyme B and IFN-γ.

**Conclusion:**

Combining the PTPN2/PTPN1 inhibitor AC484 with mosunetuzumab enhanced T-cell activation, proliferation, granzyme B and IFN-γ secretion, and anti-CLL cytotoxicity in CLL samples, supporting further evaluation of PTPN2/PTPN1 inhibition as a strategy to enhance the efficacy of CLL immunotherapy.

## Introduction

Chronic lymphocytic leukemia (CLL) is a hematologic malignancy characterized by the progressive accumulation of monoclonal, mature-appearing CD5^+^ B cells in the peripheral blood, bone marrow, and secondary lymphoid organs (1). Despite major therapeutic advances, none of the currently available therapies are curative and resistance ultimately emerges, underscoring the need for novel therapeutic strategies (2). A hallmark of CLL is profound immune dysfunction particularly affecting T cells which contributes to impaired antitumor immunity. Both CD4^+^ and CD8^+^ T cells in CLL display globally altered gene-expression profiles compared with age-matched healthy donors (3), and exhibit features of exhaustion, including diminished cytokine production, defective proliferative capacity, and impaired immune synapse formation (4). Mitochondrial dysfunction further compromises CD8^+^ T-cell cytotoxicity (5). This immune dysfunction severely limits the efficacy of immunotherapeutics that depend on competent T-cell function. CD20×CD3 bispecific antibodies like mosunetuzumab redirect T-cells to target malignant B-cells by inducing T-cell cytotoxic synapse formation independently of classical antigen recognition (6). Despite clinical success in lymphomas (7), mosunetuzumab delivers only modest benefit in CLL, primarily due to intrinsic T-cell failure rather than antibody inefficacy (8). Effective therapy thus requires strategies to reactivate the dysfunctional T-cell compartment. While approved for follicular lymphoma, mosunetuzumab is not yet FDA-approved for CLL.

Protein tyrosine phosphatase non-receptor type 2 (PTPN2) has emerged as a critical negative regulator controlling both TCR and cytokine receptor signaling. By dephosphorylating LCK and FYN, it raises the T-cell activation threshold, while also restricting cytokine-induced expansion by dephosphorylating JAK/STAT pathway components (9). PTPN2 and PTPN1 share high structural homology, particularly within their catalytic PTP domains, yet exhibit distinct contributions to T-cell cytotoxicity. Although PTPN2 has a dominant functional role, additional inhibition of PTPN1 is often required to achieve maximal immune enhancement, supporting a rationale for dual PTPN1/2 inhibition (10).

Preclinical studies show that inhibition of PTPN2/PTPN1 enhances T-cell activation, proliferation, cytokine secretion, effector differentiation, and antitumor activity (11, 12). AC484 inhibitor promotes antitumor activity through multiple mechanisms, including sensitizing tumor cells to IFN-γ, enhancing immune cell activation, and engaging CD8^+^ and NK cells. It promotes proinflammatory remodeling of the tumor microenvironment, increases TCR diversity, induces specific CD8^+^ effector T cells, prevents T-cell exhaustion, and enhances T-cell effector function (11).

This study aimed to investigate the potential of PTPN2/PTPN1 inhibition in combination with the bispecific antibody mosunetuzumab as a therapeutic approach for CLL. We hypothesized that inhibition of PTPN2/PTPN1 in CLL-derived T-cells will restore functional competence and enhance cytotoxicity against malignant B-cells, in combination with mosunetuzumab.

## Materials and methods

### Patients and samples

Cells were obtained from peripheral blood samples donated by patients fulfilling the standard criteria for CLL after signing informed consent approved by the Tel-Aviv Sourasky Medical’s Institutional Review Board according to the Helsinki Accords. Peripheral blood mononuclear cells (PBMC) were isolated by Ficoll density-gradient centrifugation. Viable frozen cells were kept in FCS containing 10% DMSO and stored in liquid nitrogen. Before use, frozen cells were thawed and cultured at 37 °C, 5% CO_2_, in RPMI medium supplemented with 10% FCS, penicillin-streptomycin and L-glutamine. The samples used contained more than 90% CLL cells. Patient characteristics are presented in **Supplementary Table 1**.

### Antibodies and reagents

PTPN2/PTPN1 inhibitor ABBV-CLS-484 (AC484,Osunprotafib,HY-145923), mosunetuzumab (HY-P99033) and human IgG kappa isotype control (HY-P99001) were from MedChemExpress (USA). Dynabeads Human T-Activator CD3/CD28 (Dy-11131D) were from Thermo Fisher Scientific (MA, USA). Ficoll-Paque PLUS from GE healthcare (Uppsala, Sweden), dimethyl sulfoxide (DMSO) from Merck (Darmstadt, Germany), RPMI, fetal calf serum (FCS), L-glutamine and penicillin-streptomycin from Biological Industries (Beit-Haemek, Israel) were utilized for cell cultures.

### Enzyme-linked immunosorbent assay

For evaluating the Granzyme B and IFN-γ protein expression level, ELISA was performed (cat. no. DY2906–05 and DY285B-05, R&D Systems, Minneapolis, MN, USA), according to the manufacturer’s instructions.

### Flow cytometry

Peripheral blood mononuclear cells were stained with Pacific Blue Anti-Human CD8 or CD4 (BioLegend) and PE Anti-Human CD69 or CD25 (BD Biosciences, CA, USA). Isotype control was PE Mouse IgG1, κ (BD Biosciences, CA, USA). Cells were washed and then suspended in 0.5 mL PBS/1% FCS. Samples were acquired by BD FACSCanto II and analyzed using BD FACSDiva software.

### Immunofluorescence

CLL cells (10 x 10^6^ /mL) were seeded on poly-L-lysine coated glass, then treated with 1µM AC484 for 2 hours, at 37 °C in a humidified 5% CO_2_ atmosphere. DMSO treated cells served as controls. Following treatment, 1μg/mL of the CD20XCD3 bispecific antibody mosunetuzumab or human IgG kappa isotype control, was added for 72 hours. The cells were fixed with 4% methanol-free paraformaldehyde for 20 min, followed by permeabilization with 0.2% TX-100 (Sigma Aldrich, MO, USA). The antibodies used were Mouse anti Human CD8 (BD Biosciences, CA, USA) (1:200) and Rabbit anti Human Granzyme B (Cell Signaling Technology (Beverly, MA) (1:300) overnight at 4 °C. FITC conjugated anti-IgM (Bethyl, TX, USA) (1:200), Anti-mouse IgG (H+L),F(ab’)2 fragment (Alexa Fluor 647 conjugate) (1:250) and Anti-rabbit IgG (H+L),F(ab’)2 fragment (Alexa Fluor 555 conjugate) (1:500) (Cell Signaling Technology, Beverly, MA), were used for 60 min at RT. After incubation, cells were washed three times with 1% BSA in PBS. All the cells were labeled with fluorescent mounting medium with DAPI (Golden Bridge International Inc., WA, USA). Images were acquired using Stellaris Leica confocal microscope (Gray Faculty of Medicine and Health Sciences core facility, Tel Aviv University).

### Cytotoxicity and proliferation assays

B and T cells were isolated from CLL patients-derived PBMCs using CD19 microbeads (130-050-301), B-CLL (130-103-466) and pan T cells (130-096-535) isolation kits (Miltenyi Biotec) according to the manufacturer’s instructions. Isolated B cells were labeled with BioTracker 488 Green CFSE dye (SCT110, Sigma-Aldrich, MO, USA). B-CLL cells and autologous T-cells (purity >95%) were seeded at 1:1 E:T ratio and incubated with AC484 for 2 hours in 96-well flat-bottom plates. DMSO treated cells served as controls. Then, 1μg/mL of the anti-CD20Xanti-CD3 bispecific antibody mosunetuzumab or an isotype control was added. Incucyte^®^ Cytotox Red Dye (4632, Sartorius) was used for counting dead cells. CLL cell killing (% green + red cells/green cells) and T cells proliferation (low green (T cells) object count normalized to time ‘0’) were analyzed using Incucyte™ system for indicated times. Nine images from distinct regions within each well were taken at intervals of 12 hours using a 20X objective. For each patient condition, cells were seeded in duplicate wells.

### Statistical analysis

To compare the two paired groups within each experiment, Student’s t-test was used for normally distributed dependent variables, and the Wilcoxon signed-rank test was used for non-parametric dependent variables. M-CLL/U-CLL subgroup analyses were considered exploratory and hypothesis-generating, and P-values were unadjusted. All statistical tests were two-sided. Statistical analyses were performed using GraphPad Prism 9 (GraphPad Software, San Diego, CA, USA). A P-value <0.05 was considered statistically significant.

## Results

First, we analyzed the effect of the PTPN2/PTPN1 inhibitor, AC484, on T-cell activation in CLL. Peripheral blood mononuclear cells (PBMCs) of patients with CLL (**Supplementary Table 1**) were treated with different concentrations of AC484, followed by incubation with anti-CD3/CD28 beads. Surface levels of the activation markers, CD69 and CD25, were determined by flow cytometry. AC484 pretreatment led to increased CD25 expression in both CD4^+^ and CD8^+^ T-cell subsets and significantly upregulated CD69 expression in CD8^+^ T cells. The optimal concentration was found to be 1 µM (**Figures 1A–D**). Subgroup analysis by immunoglobulin heavy chain gene (IGHV) mutation status revealed numerically higher levels of activation markers expression in mutated CLL (M-CLL) compared to unmutated CLL (U-CLL) group (**Figures 1E–H**).

**Figure 1:**
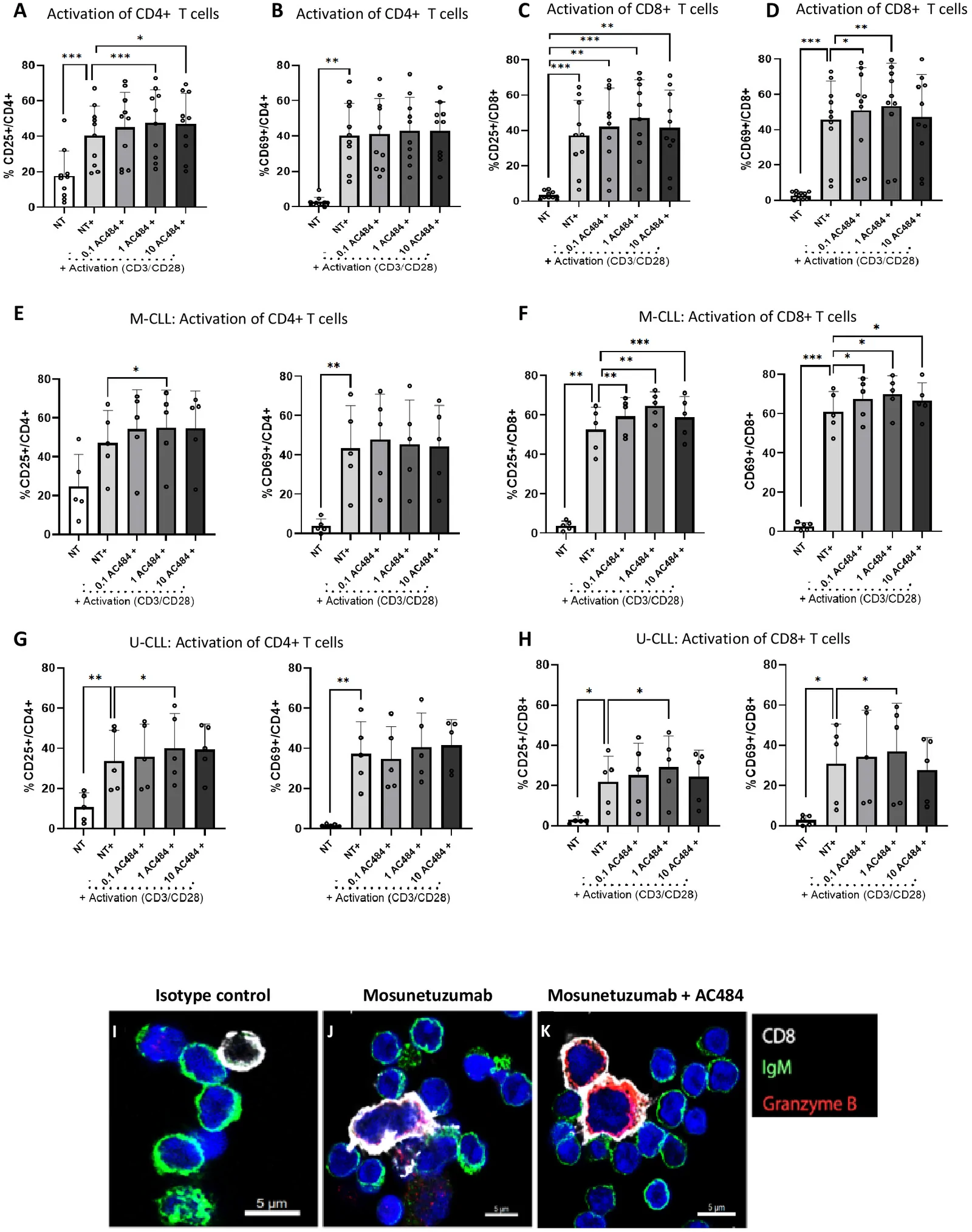
The effect of PTPN2/PTPN1 inhibition on T cells activation and synapse formation. Peripheral blood CLL cells were treated with the indicated concentration of AC484 for 2 hours. DMSO treated cells served as controls (NT**).** Following treatment, the cells were stimulated with Dynabeads^®^ Human T Cells-Activator CD3/CD28 for 24 hours at 37 °C. After incubation, the cells were stained with Pacific Blue Anti-Human CD8 or CD4 and PE Anti-Human CD69 or CD25. Samples were acquired by FACSCanto II (BD) and analyzed using BD FACSDiva software. **(A)** Quantification of %CD25+ cells in CD4^+^ population (n=10) **(B)** Quantification of %CD69+ cells in CD4^+^ population (n=10). **(C)** Quantification of %CD25+ cells in CD8^+^ population (n=10). **(D)** Quantification of %CD69+ cells in CD8^+^ population (n=10). **(E–H)** Subgroup analysis of CD25 or CD69 expression in CD8^+^ or CD4^+^ T cells population according to IGHV mutation status, **(E, F)** M-CLL (n=5) or **(G, H)** U-CLL (n=5), *p<0.05, **p<0.01, ***p<0.001. Data shown as mean+SD. **(I–K)** CLL cells were seeded on poly-L-lysine coated glass, then treated with 1µM AC484 for 2 hours. Then the cells were incubated with 1μg/mL of mosunetuzumab or human IgG kappa isotype control for 72 hours. The cells were fixed and permeabilized, followed by staining for CD8 (white), granzyme B (red), IgM (green) and DAPI (blue). Representative confocal images showing synapse formation between B cells (green) and CD8^+^ T cells (white). **(I)** Representative image of cells treated with the isotype control. **(J)** Representative image of cells treated with mosunetuzumab. **(K)** Representative image of cells treated with mosunetuzumab and the PTPN2/PTPN1 inhibitor AC484. Scale bar, 5 µm.

Next, we studied the effect of mosunetuzumab on activation of T cells in patients-derived CLL cells, especially in combination with PTPN2/PTPN1 inhibitor. A concentration of 1 µg/mL was selected based on previously published studies (13), and supported by our findings using the bispecific antibody glofitamab (14). The effect of mosunetuzumab on T-cell morphology, immune synapse formation and granzyme B induction was studied using representative confocal images. PBMCs of patients with CLL were treated with mosunetuzumab or isotype control for 72 hours, with or without pre-incubation with AC484. Then, the cells were fluorescently stained for CD8, IgM and granzyme B. In samples treated with mosunetuzumab +/- AC484 inhibitor, we observed morphological changes reflected in an increased CD8^+^ T-cell size, induction and release of granzyme B, formation of large CLL-CD8^+^ T cells clusters and strong CD8^+^ T-cell: CLL immune synapses (**Figures 1J, K**). In samples treated with isotype control, the CD8^+^ T cells were small, with almost no expression of granzyme B, and a few small cell clusters with weak CD8^+^ T-cell: CLL immune synapses were seen (**Figure 1I**).

In parallel, cytotoxicity and T-cell proliferation assays were conducted in co-cultures of purified T-cells and B-CLL cells using Incucyte™ time-lapse imaging over 7 days. PBMCs of patients with CLL were subjected to negative selection to isolate B-CLL cells and T-cells. B-CLL cells were subsequently labeled with BioTracker CFSE dye, while Cytotox red dye was used to evaluate cell death. B-CLL and T-cells were co-cultured at a 1:1 effector to target (E: T) ratio and treated with AC484 for 2 hours. Cells treated with DMSO served as controls. Following treatment, the cells were incubated with either mosunetuzumab or an isotype control. Our findings indicate a modest increase in T-cell mediated cytotoxicity (day 3) and enhanced proliferative responses following PTPN2/PTPN1 inhibition in combination with mosunetuzumab. The M-CLL group showed more pronounced differences relative to the U-CLL group. Similar to the findings observed with the confocal microscopy, the treatment also induced B-CLL/T-cell clustering (**Figures 2A–G**). In order to quantify granzyme B and IFN-γ levels we used ELISA-based assay platform. Our results indicate increased levels of granzyme B and IFN-γ in samples treated with AC484 and mosunetuzumab, with the most pronounced effect observed on day 7 (**Supplementary Figure S1**). Consequently, this time point was selected for analysis in a larger patient cohort (**Figures 2H, I**).

**Figure 2:**
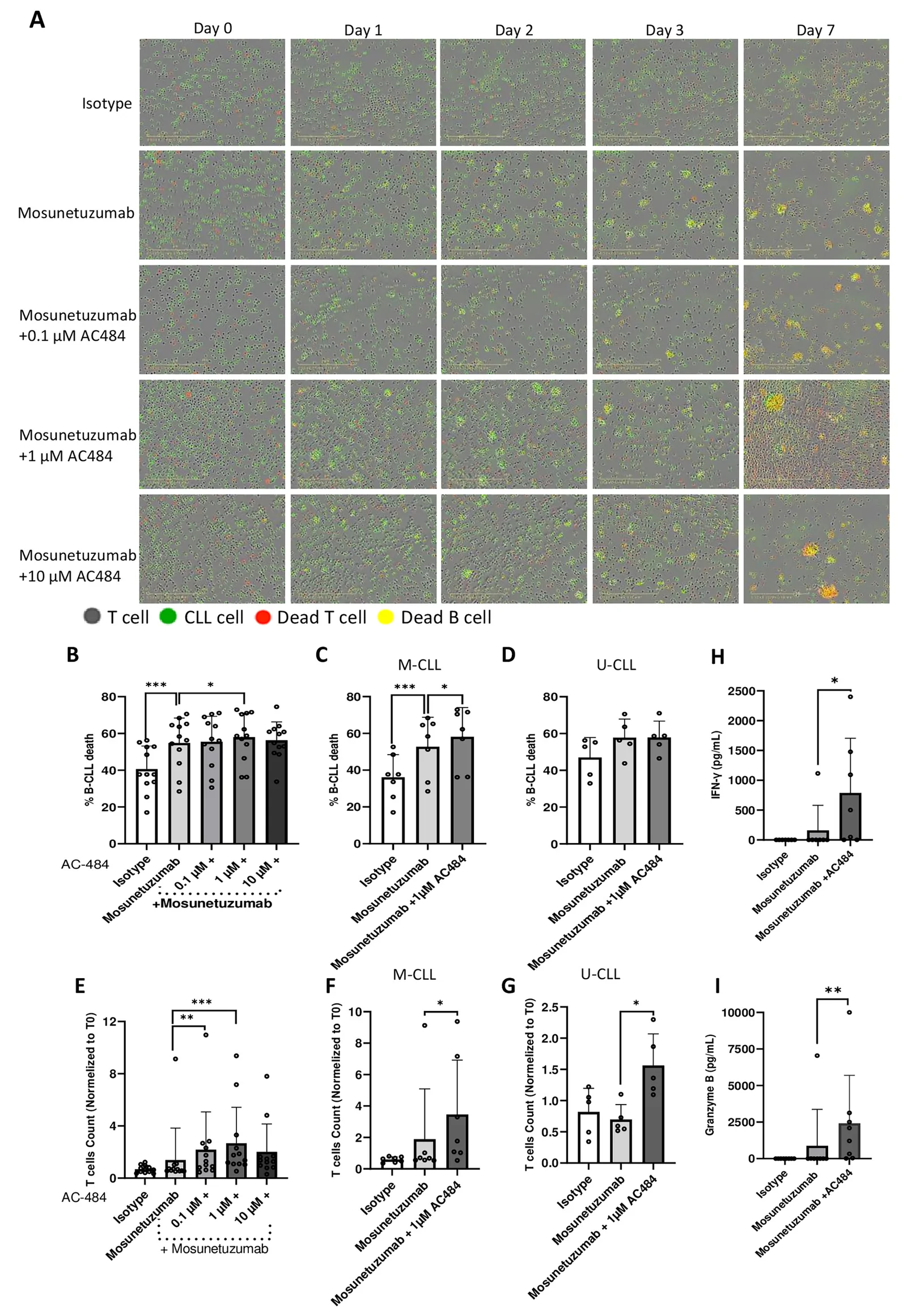
PTPN2/PTPN1 inhibition augments the cytotoxicity and proliferation of T cells in response to mosunetuzumab. B and T cells were isolated from CLL patients-derived PBMCs using B-CLL and pan T isolation kits. Isolated B cells (CLL cells) were labeled with BioTracker 488 Green CFSE dye. CLL cells and autologous T-cells were seeded at 1:1 effector-to-target (E:T) ratio and treated with the indicated concentrations of AC484 for 2 hours. Then, the cells were incubated with 1μg/mL of mosunetuzumab or isotype control for 7 days. Cytotox red dye was used to detect cell killing. CLL cell killing (% green+ red cells/green cells) was analyzed using Incucyte™ system for indicated times. T cell count was calculated as low green object count normalized to the start point. 9 images from distinct regions within each well were taken at intervals of 12 hours. The experiment was performed in duplicate. **(A)** Representative images from day 0–3 and 7 using cells from one patient. **(B)** Quantification of % CLL cell killing using mosunetuzumab (+/- PTPN2/PTPN1 inhibitor) or isotype control in 72hr (n=12). **(C, D)** Subgroup analysis of % CLL cell killing according to IGHV mutation status, **(C)** M-CLL (n=7) or **(D)** U-CLL (n=5). **(E)** Quantification of T cell proliferation at day 7 (n=12). **(F, G)** Subgroup analysis of T cell proliferation, **(F)** M-CLL (n=7) or **(G)** U-CLL (n=5). **(H, I)** Supernatants were collected on day 7, and levels of IFN-γ **(H)** and granzyme B **(I)** were quantified using an ELISA-based assay platform (n=8), *p<0.05, **p<0.01, ***p<0.001. Data shown as mean+SD.

## Discussion

In summary, this study demonstrates the potential therapeutic benefit of combining PTPN2/PTPN1 inhibition with the bispecific antibody mosunetuzumab to enhance antitumor T-cell activity in patient-derived CLL samples. In our initial experiments, T-cells from CLL patient-derived samples treated with AC484 and activated with anti-CD3/CD28 beads exhibited increased expression of surface activation markers, consistent with enhanced TCR signaling upon PTPN2/PTPN1 blockade. These observations align with prior reports showing that PTPN2/PTPN1 inhibition or deletion enhances LCK phosphorylation and downstream STAT5 signaling, thereby augmenting T-cell activation (11, 12, 15). Furthermore, the combination of AC484 with mosunetuzumab induced morphological changes compatible with T-cell activation and immune synapse formation, a process known to be defective in T-cells of CLL patients (4). This combination also enhanced T-cell proliferation, increased granzyme B and IFN-γ release, and caused a modest increase in anti-CLL T-cell cytotoxicity.

Consistent with these observations, PTPN2/PTPN1 blockade has previously been shown to enhance the antitumor activity of CAR-T cells and reverse T-cell exhaustion in preclinical solid tumor models, leading to improved antitumor immune responses *in vivo* (15, 16). Our findings further suggest that PTPN2/PTPN1 inhibition may also have potential applications for improving CAR-T-cell function and proliferation in patients with CLL. Clinically, AC484 is currently being evaluated in a Phase 1 clinical trial (ClinicalTrials.gov identifier: NCT04777994) both as monotherapy and in combination with anti-PD-1 therapy in patients with advanced solid tumors.

Overall, these findings indicate that PTPN2/PTPN1 inhibition can enhance ex vivo T-cell responses to the CD20×CD3 bispecific antibody mosunetuzumab in CLL and warrant further investigation of this combination as a potential therapeutic approach.

## Data Availability

The raw data supporting the conclusions of this article will be made available by the authors, without undue reservation.
