# Supplementary material for: PTPN2/PTPN1 inhibition enhances ex vivo T- cell responses and potentiates mosunetuzumab activity in chronic lymphocytic leukemia
Source: Front Immunol. 2026 Oct 8;17:1948967. doi: 10.3389/fimmu.2026.1948967 (PMC13687270; doi:10.3389/fimmu.2026.1948967)
Supplement: Supplementary file 1 [file DataSheet1.pdf]

Supplementary Figures

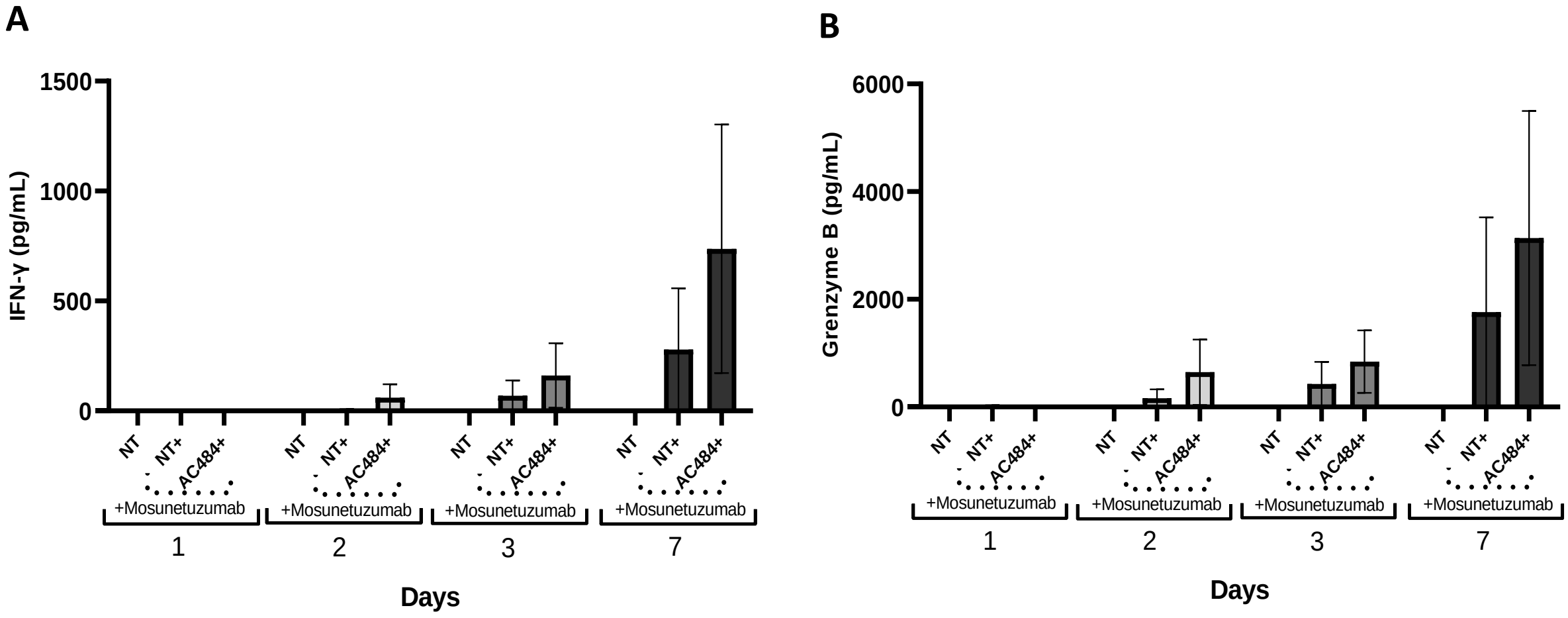

Supplementary Figure S1. Inhibition of PTPN2/PTPN1 enhances IFN-γ and granzyme B secretion in response to mosunetuzumab. B and T cells were isolated from CLL patients-derived PBMCs using B-CLL and pan T isolation kits. CLL cells and autologous T cells were co-cultured at a 1:1 effector-to-target (E: T) ratio and pre-treated with 1 µM AC484 for 2 hours. Subsequently, cells were incubated with 1 µg/mL mosunetuzumab or an isotype control for a duration of 7 days. Supernatants were collected on days 1, 2, 3, and 7, and levels of IFN-γ **(A)** and granzyme B **(B)** were quantified using an ELISA-based assay platform (n = 4).

Supplementary Table 1: Patient characteristics

Patient	Gender/Age (y)	IGHV gene SHM status	Genomic alteration	Time from diagnosis	ALC (10^3/µl)	Assay
**CLL_01**	F/58.2	M-IGHV	Normal	159 months	33.9	IF, ELISA, FACS, Incucyte
**CLL_02**	M/51.8	M-IGHV	-	59 months	36.0	FACS, Incucyte
**CLL_03**	M/68.9	M-IGHV	Dell3q	68 months	21.2	ELISA, FACS, Incucyte
**CLL_04**	F/82.0	M-IGHV	Del13q, Del11q, Del17p	292 months	71.1	FACS
**CLL_05**	F/78.3	M-IGHV	Normal	171 months	139.4	ELISA, FACS, Incucyte
**CLL_06**	M/57.2	UM-IGHV	Del11q	62 months	46.6	ELISA, FACS, Incucyte
**CLL_07**	M/44.3	UM-IGHV	Del13q, Del11q	17 months	218.3	IF, FACS
**CLL_08**	F/60.4	UM-IGHV	Del13q	84 months	175.6	ELISA, FACS
**CLL_09**	F/71.1	UM-IGHV	Tri12	102 months	183.7	FACS
**CLL_10**	F/76.8	UM-IGHV	Del13q	90 months	203.1	FACS
**CLL_11**	M/55.5	M-IGHV	Normal	55 months	48.3	ELISA, Incucyte
**CLL_12**	F/76.2	M-IGHV	Del13q	194 months	100.2	Incucyte
**CLL_13**	M/59.7	M-IGHV	Del13q	121 months	116.4	ELISA, Incucyte
**CLL_14**	F/65.5	UM-IGHV	Del13q, Tri12	76 months	131.1	Incucyte
**CLL_15**	F/75.1	UM-IGHV	Del13q, Del11q	205 months	77.0	Incucyte
**CLL_16**	F/40.1	UM-IGHV	Del13q	49 months	79.3	Incucyte
**CLL_17**	M/71.9	UM-IGHV	Del11q	41 months	158.0	Incucyte
**CLL_18**	M/67.0	UM-IGHV	Normal	68 months	113.2	IF
**CLL_19**	M/72.8	M-IGHV	Del13q	254 months	22.1	ELISA
**CLL_20**	M/77.1	M-IGHV	Del13q, Del11q	290 months	61.9	IF

M-male, F-female, y- years, M-IGHV- mutated IGHV, UM-IGHV-unmutated IGHV, ALC- absolute lymphocyte count, IF- Immunofluorescence.
